# Extracorporeal membrane oxygenation improves coagulopathy in an experimental traumatic hemorrhagic model

**DOI:** 10.1007/s00068-016-0730-1

**Published:** 2016-11-04

**Authors:** M. Larsson, P. Forsman, P. Hedenqvist, A. Östlund, J. Hultman, A. Wikman, L. Riddez, B. Frenckner, M. Bottai, C.-M. Wahlgren

**Affiliations:** 1 Department of Molecular Medicine and Surgery, Karolinska Institutet and University Hospital, SE-171 76 Stockholm, Sweden; 2 ECMO Department Karolinska University Hospital, Karolinska Institutet and University Hospital, SE-171 76 Stockholm, Sweden; 3 Department of Vascular Surgery/Trauma Center, Karolinska Institutet and University Hospital, SE-171 76 Stockholm, Sweden; 4 Department of Anesthesiology and Intensive Care, Karolinska Institutet and University Hospital, SE-171 76 Stockholm, Sweden; 5 Department of Clinical Immunology and Transfusion Medicine, Karolinska Institutet and University Hospital, SE-171 76 Stockholm, Sweden; 6 Department of Clinical Sciences, Swedish University of Agricultural Sciences, SE-750 07 Uppsala, Sweden; 7 Unit of Biostatistics, Institute of Environmental Medicine, Karolinska Institutet, Stockholm, Sweden

**Keywords:** Trauma, Shock, Coagulopathy, Animal model, Extracorporeal circulation, ECMO, Hemorrhage, Resuscitation

## Abstract

**Purpose:**

Hemorrhage is the most common cause of preventable death after trauma. Coagulopathy plays a central role in uncontrolled bleeding and is caused by multiple factors. Extracorporeal Membrane Oxygenation (ECMO) is an established treatment for patients with respiratory failure and has in recent years also been used in severely injured trauma patients with cardiopulmonary failure and coexisting bleeding shock. The aim of this study was to evaluate the effect of ECMO on hypothermia, acidosis, and coagulopathy in a traumatic hemorrhagic rabbit model.

**Methods:**

After anesthesia and tracheostomy, ten New Zealand White rabbits sustained laparotomy, bilateral femur fractures and were hemorrhaged 45% of their estimated blood volume. After 90 min of hemorrhagic shock they were resuscitated with a standard transfusion protocol together with venoarterial ECMO (*n* = 5) or with a standard transfusion protocol only (*n* = 5) for 60 min. No systemic heparin was administered.

**Results:**

ECMO during 60 min of resuscitation significantly increased heart rate (*p* = 0.01), mean arterial pressure (*p* = 0.01), body temperature (*p* = 0.01) and improved the metabolic acidosis, pH (*p* = 0.01), and lactate (*p* = 0.01). ECMO also improved the coagulation capacity measured in vitro by Rotational Thromboelastometry with a significant decrease in clot formation time (*p* < 0.01). This finding was confirmed in vivo with a significant reduction in the animals’ ear bleeding time (*p* < 0.01) and cuticle bleeding time (*p* < 0.01); 5/5 animals survived in the ECMO group and 3/5 animals survived in the control group.

**Conclusions:**

Heparin-free ECMO stabilizes circulation, improves coagulation, and may impact short-time survival, during the first 60 min, in an experimental traumatic model with severe hemorrhagic shock.

## Introduction

Major trauma is the leading cause of death worldwide in the young population. Hemorrhage is the most common preventable cause of death after trauma and accounts for up to 30–40% of trauma-related deaths [1–4]. Early resuscitation emphasizes adequate ventilation, damage control surgery (DCS) with rapid control of bleeding, blood transfusion, and prevention or early management of coagulopathy [5, 6].

Acute traumatic coagulopathy (ATC) is multifactorial, involves the whole process of hemostasis, and is associated with severe injury. Six key factors for initiation of ATC have been identified. They are tissue trauma, shock, hemodilution, hypothermia, acidosis, and inflammation [7]. Tissue injury needs to be combined with hypoperfusion of organs to induce ATC [8]. Acidosis is mainly the result of hypoperfusion and impairs the function of clotting factors. Hypothermia and metabolic acidosis are late effects of bleeding shock and clearly enhance coagulopathy. Acidosis and hypothermia do not seem to cause clinical relevant effects on protease function until pH is <7.2 and temperatures are under 33 °C. Platelet function and aggregation are reduced at temperatures lower than 35 °C [9–12]. Extracorporeal Membrane Oxygenation (ECMO) has been an established treatment for severe respiratory failure for more than 20 years [13, 14]. ECMO treatment in trauma patients with severe hypoxia due to pulmonary contusions or acute respiratory distress syndrome (ARDS) has previously been described [15]. In severe hemorrhage with hemodynamic compromise ECMO therapy is less well established even though occasional reports on immediate ECMO in multi-trauma have been published [16–19].

This is a proof-of-concept study and the aim was to investigate if venoarterial ECMO (VA–ECMO) could reverse acidosis, hypothermia, and coagulopathy in a traumatic hemorrhagic rabbit model during the initial 60 min of resuscitation.

## Materials and methods

### Study animals and control group

**Fig. 1:**
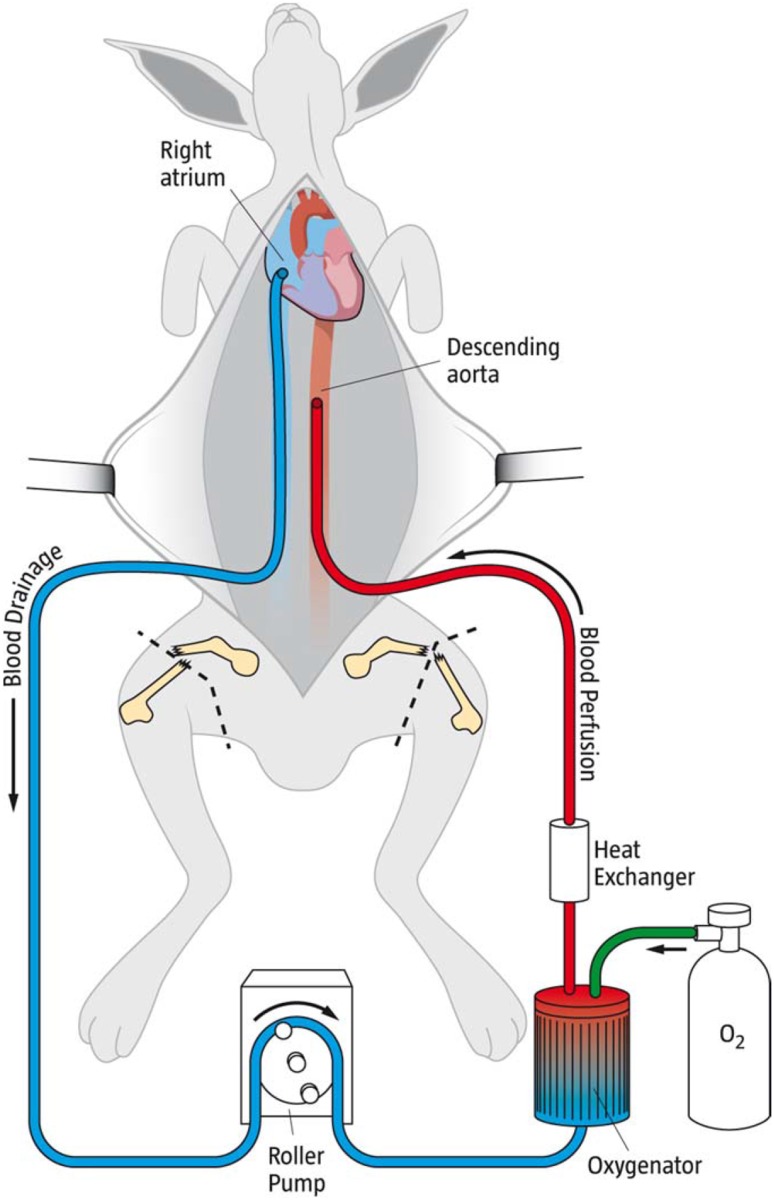
The Rabbit ECMO-Trauma model. The animal sustained laparotomy and bilateral femur fractures and were exsanguinated to class IV Shock. The venoarterial ECMO circuit: Venous draining cannulae in the right atrium (*blue*), roller pump, heparinized membrane oxygenator for saturation and carbondioxide removal, water heat exchanger (38.5 °C). Blood is reinfused in the descending aorta (*red*). Bilateral femurfractures are indicated

**Fig. 2:**
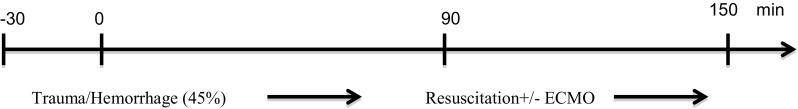
Flowchart illustrating the study’s timeline. Induction of anesthesia was at −30 min. All rabbits (n10) sustained laparotomy and femurfractures at baseline, 0 min. During 30 min blood was drained from the IVC. The animals were kept in bleeding shock for another 60 min (goals were mean arterial pressure <20 mmHg, temperature <32 °C, and pH < 7.3). At 90 min resuscitation began with a standard protocol (*n* = 5) or with a standard protocol and venoarterial ECMO in addition (*n* = 5). Rotational thromboelastometry and standard coagulation tests were controlled at 0, 90 and 150 min. The animals were killed at 150 min

15 male New Zealand White rabbits (mean weight 3.85 kg) were used in an experimental trauma model with bilateral femur fractures, laparotomy, and a class IV bleeding shock (>40% hemorrhage of blood volume) (Fig. 1). Five rabbits, the control group, were treated with a standard transfusion protocol meaning transfusion of citrated whole blood, calcium, and active rewarming. Another five rabbits, the ECMO group, received the same treatment as the former group but were also on venoarterial ECMO (VA–ECMO) for 60 min (Fig. 2). Finally, five rabbits were used as blood donors for priming the ECMO-circuits (one rabbit/circuit). All rabbits were siblings with blood group B. The ECMO model was previously developed and used [20].

### Outcomes

The primary outcomes were coagulation status, pH, lactate, base excess, and body temperature after 60 min of resuscitation. Secondary outcomes were heart rate, mean arterial pressure, and short-term survival.

### Anesthesia

For premedication medetomidine (0.2 mg/kg) was administered subcutaneously. Total intravenous anesthesia (TIVA) was performed with sufentanil (6.9 mg/kg per hour and midazolam (1.35 mg/kg per hour). Before initiation of the femur fractures, the TIVA was changed to ketamine infusion (12.5–25 mg/kg/h) for the remainder of the experiment. No neuromuscular blocking agents were used.

### Instrumentation

Percutaneous venous catheters were placed in both ears for administration of drugs. An arterial line was placed in the right ear artery for blood pressure monitoring before the hemorrhage phase in both groups and during the resuscitation phase in the ECMO group. 5 Fr introducers were placed in the inferior vena cava (IVC) and descending aorta (DA). The vessels were cannulated for blood drainage and potential connection to an ECMO system. Arterial pressure was controlled in the DA during the hemorrhage phase in both groups and during the resuscitation phase in the control group. If the animal was to be treated with VA-ECMO, the IVC was cannulated with a 12 Fr Fem-Flex II venous cannula (Edwards Lifesciences™) with the tip in the right atrium of the heart. The DA was cannulated with the tip pointing towards the heart with an 8 Fr Fem-Flex II arterial cannula (Edwards Lifesciences™) (Fig. 1).

### Ventilation and ECMO

The animals were tracheotomized with a 3.5 mm cuffed endotracheal tube and put on pressure controlled mechanical ventilation with 25–30% oxygen, 8–15 breaths/min with 4 cmH_2_O post end expiratory pressure (PEEP) and peak pressure of 20 cmH_2_O (Servo 900 C^®^, Siemens-Elema). Repeated blood gas analyses were performed (ABL 800, Radiometer) and ventilation was adjusted accordingly aiming for normoventilation before trauma was induced.

The ECMO circuit was custom-made and consisted of a Stöckert^®^ roller pump, a Medos^®^ Hilite LT Infant 800 oxygenator and ¼” surface-heparinized standard tubings of 200 cm length. The blood was warmed 38.5 °C using a water-filled heat exchanger (HICO-Aquaterm 660, Hirtz). The ECMO systems were primed with 120 ml of citrated blood from the donor rabbits. Citrate ratio: 1 part sodium citrate 2.63 g/L (Macopharma^®^)/12 parts blood). No heparin was added and no transfusion reactions were observed. Sweep gas flow over the oxygenator was set on O_2_ 1000 ml/min and CO_2_ 50 ml/min, to inhibit elimination of CO_2_ over the oxygenator (Fig. 1).

### Monitoring

The animals were monitored continuously with electrocardiogram (ECG); mean arterial pressure (MAP), esophageal temperature, saturation of the tongue and end-tidal CO_2_.

### Traumatic hemorrhage phase

The rabbits initially sustained midline laparotomies and bilateral femur fractures. The fractures were located centrally on both femurs using two small pipe wrenches with a distance of 2 cm. By fixating both ends of the femurs with the pipe wrenches and moving the wrenches shafts apart, mid-shaft femur fractures were induced on both sides of each study animal. 45% of the rabbits estimated blood volume (70 ml/kg) was drawn with syringes from the IVC continuously during 30 min. The drained blood was citrated (ratio 1/12) and stored in a blood warmer at 38.5 °C. The animals were kept in hemorrhagic shock for an additional 60 min. Ringer’s solution was infused to keep the MAP at approximately 20 mmHg, a temperature of less than 32 °C and a pH below 7.3. The total hemorrhagic shock time was 90 min (Fig. 2).

### Resuscitation phase

After the traumatic hemorrhage shock phase, the resuscitation phase started. The control group was transfused during 30 min with warm citrated blood (38.5 °C), followed by rewarming with a heating mattress for another 30 min. If calcium levels fell below 1.0 mmol/L, calcium was substituted (Calcium Sandoz^®^).The ECMO group received the same treatment as the control group and VA-ECMO with a flow rate of 100 ml/kg/min for 60 min, in addition (Figs. 1, 2). At the end of the experiment (150 min) all animals were killed in deep anesthesia with Pentobarbitalnatrium^®^ 120 mg/kg (Fig. 2).

### Blood chemistry and coagulation status

Blood-gasses were drawn every 15 min and analyzed immediately for pH, pCO_2_, pO_2_, Base Excess (BE), Hemoglobin, Hematocrit, K^+^, Na^+^, Ca^2+^, Cl^−^, Glucose, and Lactate.

At baseline (0 min), after 90 min of hemorrhagic shock and after 60 min of resuscitation, or when it was obvious that asystole was to occur within minutes, the following coagulation tests were controlled—activated partial thromboplastin time (aPTT), D-dimer, fibrinogen concentration, fibrin, prothrombincomplex(PT)/international normalized ratio (INR), platelet count (PLT), and ROTEM.

### Rotem

The ROTEM^®^ analyses were performed on whole blood, collected in tubes containing 0.129 mol l^−1^ sodium citrate and analyzed according to manufacturer’s instruction ROTEM^®^ Delta 3000 (TEM innovations, GmbH, München, Germany). Analyses were performed within 30 min at a temperature adjusted to the actual rabbit’s body temperature. The variables analyzed in ROTEM were extrinsic pathway (EXTEM) and intrinsic pathway (INTEM). Clotting time (CT) reflected the time until initiation of a clot. Clot formation time (CFT) reflected the time until a 4 mm clot was formed. EXTEM maximum clot firmness (MCF) reflected the stability of the clot. Fibrinogen function (FIBTEM) and heparin effect (HEPTEM) were also analyzed.

### Bleeding time

In vivo bleeding times were controlled at baseline, at the end of resuscitation or prior to asystole. Bleeding time was controlled as follows. The animals’ Ear Bleeding Time (EBT) was tested with Surgicut ^®^ Jr (SUJ 50i) and blotting paper in both ears [21]. The cuticle bleeding time (CBT) was also measured with a standardized incision in the second digit of the right front foot by cutting the claw with a sharp nail clipper, including the apex of the cuticle, thereby inducing a combined arterial and venous bleeding [22].

### Statistical analyses

The statistical analyses were made by the Biostatistics Core Facility, Karolinska Institutet. All the outcome variables described in the above subsections were considered. The outcome variables were analyzed one at a time. Each outcome variable was compared between the two treatment arms over the study follow-up period from baseline to 150 min. For each outcome variable, the mean value was estimated over follow-up time and by treatment arm with a linear regression model that included minutes since intervention as categorical variable, the binary indicator for treatment, and all pair-wise interaction terms. The standard errors were obtained with a cluster robust estimator that took the within animal dependence into account [23]. The animals represented the clusters. The estimated means and 95% confidence intervals are reported in the figures. The differences between means of outcome variable at 90 min and at 150 min were obtained from the robust regression models. In addition, the distribution of each outcome variable at 90 min and 150 min was tested with the Wilcoxon’s rank-sum test.

## Results

### Traumatic hemorrhage phase

**Fig. 3:**
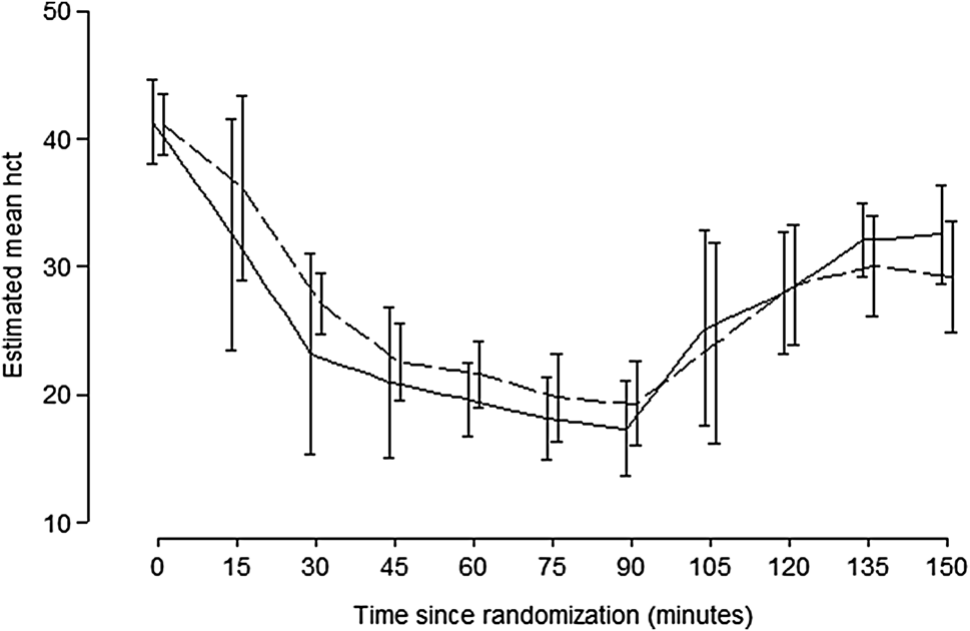
The Hematocrit during the study. The exsanguination and transfusion in the two groups were equal. Control (*solid line*) ECMO (*dashed line*)

After traumatic injury and 90 min of hemorrhagic shock, all animals (*n* = 10) were in a critical state with bradycardia (<100 bpm) and fulfilled the set criteria of hypotension (MAP ≤ 20 mmHg), hypothermia (*T* < 32 °C), metabolic acidosis (pH < 7.3), and coagulopathy (ROTEM clot amplitude <35 mm at 5 min). There was no significant difference in acidemia (pH, pCO_2_, BE, lactate), temperature, hemodynamic status (HR and MAP) or ROTEM between the two groups after the 90 min of traumatic hemorrhage. The hematocrit did not differ between the two groups after hemorrhage and resuscitation (Fig. 3).

### Resuscitation phase

#### Hemodynamic status

**Fig. 4:**
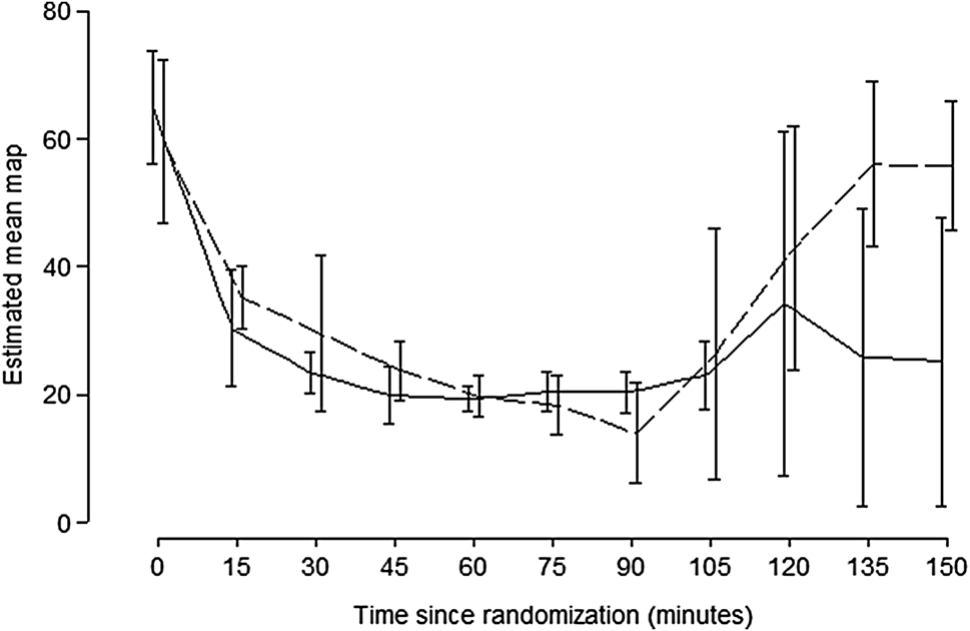
The animals’ mean arterial pressure during the study. At 90 min after hemorrhagic shock there was no difference in mean arterial pressure between the two groups but after 60 min of resuscitation the mean arterial pressure in the ECMO group was significantly improved (*p* = 0.01 respectively). Control (*solid line*) ECMO (*dashed line*)

Immediately after initiation of ECMO, the HR and MAP improved and after 60 min the animals were close to a normal circulatory state. After resuscitation in the control group the HR and MAP increased during the first 30 min. MAP never exceeded 40 mmHg and then decreased again. There was a significant difference in MAP, favoring the ECMO group, after 60 min of resuscitation (Fig. 4). Although no specific immune testing of the blood was performed, no signs of adverse effects of the allogeneic blood transfusions were observed.

#### Ventilation and ECMO

The animals were equally saturated in both groups during baseline and hemorrhage. After resuscitation and before circulatory arrest pO_2_ in the control group was above normal (mean 27.0 kPa) due to increased oxygen in the ventilator. No arterial blood gas was performed in ECMO group due to a lack of central arterial access and no AV-bridge in the circuit. Instead pre-oxygenator blood gas was drawn reflecting the rabbit’s respiratory status and acid–base status. The pCO_2_ (Table 1) was equal in the two groups, both at the end of the hemorrhage phase and after resuscitation. A limited stable ECMO flow of 100 ml/min was obtained.

**Table 1:** Blood and coagulation

	Baseline, 0 min	Control, 90 min	ECMO, 90 min	*P* ^1^	Control, 150 min	ECMO, 150 min	*P* ^2^
Temp (°C)	38.4 ± 0.1	30.3 ± 0.4	30.7 ± 0.2	0.34	29.3 ± 0.4	37.3 ± 0.3	0.01
pH	7.44 ± 0.01	7.20 ± 0.04	7.16 ± 0.10	0.38	6.90 ± 0.03	7.08 ± 0.04	0.01
pCO_2_ (kPa)	7.11 ± 0.25	5.90 ± 0.89	6.13 ± 0.64	0.75	7.02 ± 0.93	7.48 ± 0.54	0.46
Lactate (mM)	1.07 ± 0.05	11.6 ± 1.4	8.9 ± 1.1	0.10	14.3 ± 1.1	7.8 ± 0.7	0.01
BE (mM)	2.79 ± 0.03	−12.3 ± 1.8	−14.3 ± 2.3	0.26	−21.4 ± 0.5	−16.2 ± 1.0	0.01
Hb (g/L)	134 ± 3	56 ± 4	62 ± 3	0.17	90 ± 16	93 ± 8	0.45
PLT × 10^9^/l	193 ± 17	131 ± 23	93 ± 18	0.17	149 ± 20	85 ± 17	0.02
aPTT (s)	14.4 ± 2.6	20.6 ± 4.3	17.7 ± 2.7	0.16	29.0 ± 87	11.0 ± 23	0.07
INR	0.9 ± 0.0	1.3 ± 0.15	1.74 ± 0.33	0.20	1.36 ± 0.09	1.44 ± 0.2	0.42
Fibrinogen (g/l)	2.2 ± 0.0	1.2 ± 0.3	0.9 ± 0.2	0.38	1.3 ± 0.2	1.0 ± 0.2	0.38
CT_Ext_ (s)	45 ± 2	69 ± 4	764 ± 687	0.04	827 ± 710	79 ± 8	0.17
CFT_Ext_ (s)	42 ± 1	170 ± 14	1141 ± 965	0.46	1770 ± 1001	111 ± 19	0.01
MCF_Ext_ (mm)	66.7 ± 0.5	51.2 ± 0.9	42.2 ± 10.4	0.30	35.6 ± 10.6	51.6 ± 2.8	0.20
CT_Int_ (s)	181 ± 28	262 ± 357	245 ± 64	0.46	948 ± 693	290 ± 93	0.34
MCF_Fib _(mm)	11.3 ± 0.5	4.0 ± 0.3	4.2 ± 1.0	0.33	3.4 ± 1.0	4.8 ± 1.0	0.19
EBT (s)	66.0 ± 9.0	–	–	–	162.0 ± 21.0	55.5 ± 7.0	0.01
CBT (s)	70.5 ± 10.5	–	–	–	174.0 ± 15.0	75.0 ± 13.0	0.01

Baseline represents all animals. The control and ECMO groups are compared after hemorrhagic shock (90 min) and after resuscitation (150 min) and* p* values are calculated with Rank-Sum Test. *P* ^1^ 90 min, *P* ^2^ 150 min Values presented as Means with SEM

*Temp* temperature, *BE* base excess, *Hb* hemoglobin, *PLT* platelets, *aPTT* activated partial thromboplastin time, *INR* international normalized ratio (a Ratio of Prothrombine Time), *CT*_*Ext*_ clotting time extrinsic, *CFT*_*Ext*_ clot formation time extrinsic, *MCF*_*Ext*_ maximum clot firmness extrinsic, *CT*_*Int*_ clotting time intrinsic, *MCF*_*Fib*_ maximum clot firmness fibtem, *EBT* ear bleeding time, *CBT* cuticle bleeding time

#### Acid–base balance

**Fig. 5:**
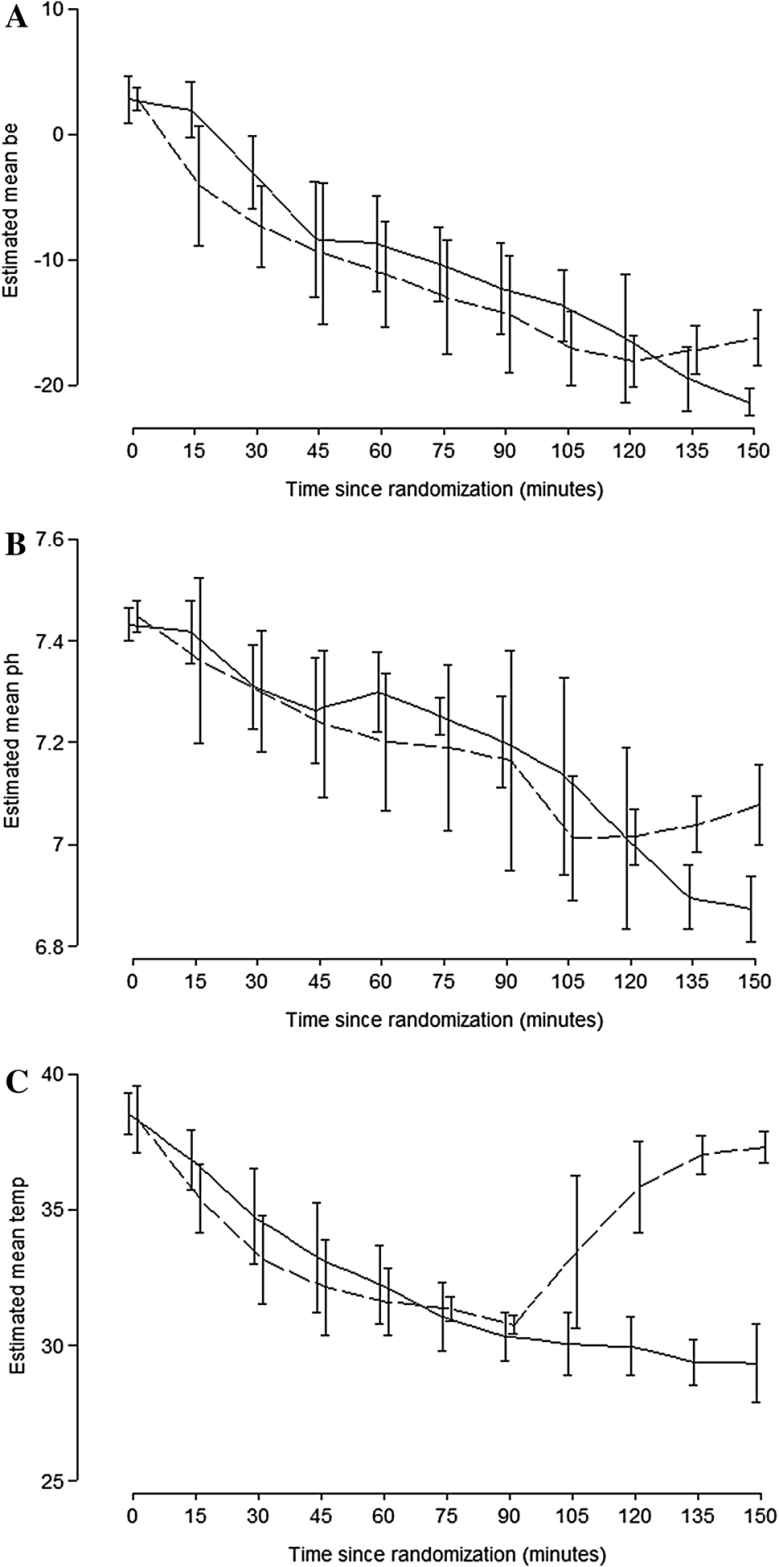
**a, b** The animals acid–base balance during the study. 90 min after hemorrhagic shock there was no difference in Base Excess (**a**) or pH (**b**) between the two groups. After 60 min of resuscitation the Base Excess and the pH were significantly higher in the ECMO group (*p* = 0.01, respectively). Control (*solid line*) ECMO (*dashed line*). **c** The animals’ body temperature. Initiation of ECMO increased the animals body temperature efficiently and after 60 min the animals in the ECMO group were significantly warmer (>37 °C) than the controls (*p* = 0.001)

There was a significant fall in pH, decrease in BE, and rise in lactate, in both groups during the traumatic hemorrhage phase (Fig. 5a, b; Table 1). The lactate concentration slowly started to fall with ECMO but continued to rise in the control group after resuscitation. At the end of the resuscitation phase lactate was significantly lower in the ECMO group. After 15 min of ECMO, pH started to increase and was over 7.0 after 60 min. Meanwhile the pH continued to deteriorate in the control group. The difference in pH between the two groups after 60 min reached statistical significance. Base Excess started to increase after 30 min of ECMO treatment and was overall significantly improved compared to the control group.

#### Body temperature

ECMO significantly improved the animal’s body temperature and after 60 min of active warming the temperatures were almost normalized (>37 °C). The body temperatures remained low (below 30 °C) in the control group (Fig. 5c).

#### Coagulation in vitro (Table 1)

The contact pathway of coagulation was evaluated with aPTT and ROTEM Clotting Time, CT_Intem_. The aPTT decreased in the ECMO group during the 60 min of resuscitation, while aPTT, in the control group, continued to rise. The difference between the two groups did not reach statistical significance. CT_Intem_ increased in both groups, but even though there was no significant difference at the end of resuscitation, the rise was higher in the control group.

The tissue factor dependent extrinsic pathway of coagulation was analyzed by INR, ROTEM CT_Extem,_ and CFT_Extem_. INR decreased after 60 min of ECMO while it remained on the same level in the control group.

The extem clotting time (CT_Extem_) was considerably reduced in the ECMO group, while it was largely prolonged in the control group and there was a difference between the groups at the end of the experiment. The clot formation time (CFT_Extem_) followed the same pattern and was significantly reduced in the ECMO group after resuscitation.

Analyzing the clot firmness with ROTEM: MCF_Extem_ (reflecting both platelet and fibrinogen contribution) was slightly increased after ECMO for 60 min. while it was reduced in the control (*p* = 0.2). The MCF_Fibtem_ (only fibrinogen contribution) showed low detectable values at the end of the experiments equally in both groups. The Fibrinogen levels were also equally diminished in the two groups and did not increase after transfusion.

Neither in the study group nor in the control group, did the ROTEM curves show any fibrinolysis. Analyses with HEPTEM, blocking the heparin effect, did not change the INTEM CT or CFT variables. This indicates that the systemic effect of surface heparin was negligible.

The platelet count was equal in the two groups initially but after 90 min of hemorrhagic shock the platelet count was lower in the ECMO group. The number of circulating platelets kept falling and was at the end of the resuscitation phase significantly reduced in the ECMO group compared to the control group.

#### Coagulation in vivo (Table 1)

The baseline bleeding times according to EBT and CBT were equal in the two groups. After resuscitation both the difference in EBT and CBT were statistically significant, favoring the ECMO group.

#### Outcome and survival

In the ECMO group, three animals (3/5) were asystolic and one was severely bradycardic (HR < 10) at cannulation. They all received external cardiac compressions and recovered with recapture of sinus rhythm and normalized blood pressure after initiation of extracorporeal circulation. In summary, all animals (5/5) in the ECMO group survived with stabilized vital status and improved coagulation. Two animals (2/5) in the control group were asystolic, 28 min, respectively, 30 min after initiation of resuscitation (transfusions were completed in both cases). They received external cardiac compressions but did not regain sinus rhythm. The surviving animals in the control group (3/5) were all severely hypotensive and hypothermic.

## Discussion

Trauma-induced coagulopathy is an important factor for hemorrhagic death in trauma victims [7]. When fully developed, the coagulopathy is very difficult to reverse. This study shows that VA-ECMO improves acidosis and temperature in a traumatic experimental hemorrhagic model. The heater in the ECMO circuit was superior compared to warm transfusions and heating mattress in the control group. ECMO increased the temperature above 37 °C and increased pH above 7.0 within 60 min. ECMO also improved central circulation in terms of blood pressure and heart rate. When assisted by VA-ECMO all the animals in severe hemorrhagic shock survived. The extrinsic coagulation pathway reflected by clot formation time (CFT_Extem_) was improved and there was a trend of improvement in the intrinsic pathway as well. In vivo testing of the coagulation capacity with ear bleeding and cuticle bleeding times showed an improvement in the ECMO group.

To our knowledge no similar study has previously been performed. This rabbit ECMO model was earlier used evaluating the Factor XII antibody 3F7 [20]. ECMO has previously been used in a rabbit-trauma model where ECMO-resuscitation for prolonged hemorrhagic shock (3 h) improved tissue perfusion, reduced systemic inflammation, and alleviated the intestinal damage that may be one important factor for Multi Organ Failure (MOF) [24]. Park et al. showed that rats exposed to both hemorrhagic shock and hypothermia have a prolonged clot formation time and reduced MCF [25]. The former finding is verified by the present results, but ECMO only slightly improved the clots firmness. This may be due to the consumption of fibrinogen. The fibrinogen levels were equally reduced after hemorrhagic shock in both groups and the ECMO system did not seem to aggravate fibrinogen consumption.

Apart from a lower platelet count and much higher CT_Extem_ and CFT_Extem_ in the ECMO group after hemorrhagic shock there was no difference between the groups before resuscitation.

In spite of the initial advanced coagulopathy, several coagulation parameters improved after ECMO-resuscitation. Engstrom et al. showed the importance of an increased pH and reduced lactic acidosis to avoid impairment of the coagulation cascade in two previous studies [12, 26]. We believe that the improvement of tissue perfusion with ECMO resulted in a gradual normalization of pH and lactate. This may have had a beneficial effect on the coagulation capacity. To gain efficiently strong clots after this type of trauma it is necessary to substitute fibrinogen. In the present study the fibrinogen was consumed, stayed on a low level and was not substituted. This was verified by a reduction of the maximum clot firmness. These findings suggest that the reduction was mainly due to low fibrinogen levels. In a clinical setting fibrinogen would have been substituted.

Extracorporeal devices activate the coagulation system [27] and previous studies have shown decreased numbers of circulating platelets in extracorporeal devices [28, 29]. This finding was confirmed in the present study. The number of circulating platelets did not reach levels below 50x10^9^/L and the clotting ability did not seem to be negatively affected. ECMO in trauma has been somewhat controversial but has in recent years gained popularity both in the post-trauma ARDS situation and in hypovolemic shock during the early resuscitation phase [16]. In our clinical experience, we have successfully resuscitated selected patients with respiratory failure and concomitant hemorrhagic shock due to thoraco-abdominal trauma with venoarterial ECMO (unpublished data). In this model no intentional pulmonary injury was induced. A high pO_2_ in the control group supports this. ECMO improves both hypoxic metabolic acidosis and hypercapnic respiratory acidosis. In combination with lung injury ECMO may add further positive effects on the traumatic coagulopathy. An earlier experimental study described how VA-ECMO reduced systemic venous pressure while maintaining or improving systemic perfusion in both a normal circulatory state and in the setting of increased right ventricular load associated with acute lung injury [30]. ECMO may theoretically be a useful tool in reducing blood loss during major venous hemorrhage, but other positive or negative effects of ECMO in trauma need to be investigated.

There have been concerns about using heparin in trauma patients on ECMO. We did not use systemic heparin as anticoagulant for the ECMO system but heparinized surfaces of tubings and oxygenators. The blood used for priming the circuit was citrated with the same concentration as blood products that are normally transfused to trauma victims. Calcium was substituted as needed. Viscoelastic heparin test did not show any effect of free heparin in the system. The aPTT were reduced during ECMO treatment but there were no signs of clotting in the circuits. In the future it may be possible to use anticoagulant drugs that do not affect the bleeding risk [20].

Inflammation and coagulation are largely activated in ECMO systems [31]. In this study we did not focus on the immune system or inflammation. The aim was to study the effect of ECMO support on hypothermia, acidosis, and coagulopathy in the first initial 60 min of trauma resuscitation. The ability of ECMO to fast increase the body temperature during this time is obvious but the reduction of lactate and improvement of pH is somewhat slower. If ECMO had been extended for more than 60 min or even continued for the first 24 post-trauma hours, the acid–base balance might have been improved further. ECMO in trauma may be performed without heparin for at least 24 h.

A low ECMO flow was accepted due to limited drainage. This limitation was caused by hypovolemia and in this study a lack of further transfusion possibilities. In clinical trauma scenarios, continuous bleeding is a common problem which can cause difficulties to withhold a high ECMO flow. However, with the availability of donor blood, the ECMO circuit can quickly be filled with more blood, and the large bore cannulas are optimal for high volume infusion [16].

There are some obvious limitations with our study. The number of study animals is small, especially as in the control group at the end of the study only three animals are left. The posttraumatic observation time was short, only 150 min. The small animal model with rabbits was used because of earlier experience, but a large animal model may have been closer to a human scenario. The coagulation system in rabbits is slightly different compared to humans but their coagulation system is constructed as the human with extrinsic, intrinsic and a common pathway’s and consists of the same coagulation factors. Some serin protease activities are different. Rabbits have a 50 times higher activity of factor V than humans and the activity of factor XI and factor XIII is also slightly higher. This may explain why rabbits have shorter aPTT and PT [32]. The different activity may limit implications for human management and the variations of our coagulation data may be explained by a limited time of ECMO-resuscitation. Furthermore, it is difficult to simulate a realistic traumatic model with coagulopathy in animals [33]. However, this is the first experimental pilot study showing ECMO’s effect on temperature, acidosis, and coagulation in a rabbit model of trauma and hemorrhage. Further studies to evaluate ECMO’s role in trauma are needed and indications for VA-ECMO in severe trauma need to be established in collaboration between the trauma communities and ECMO communities.

## Conclusions

VA-ECMO seems beneficial in an experimental traumatic hemorrhagic shock model for stabilization of central hemodynamics, improvement of acidosis, body temperature, and coagulopathy during the initial resuscitation phase. ECMO may increase the likelihood of survival but this need to be confirmed in larger future studies.
